# Modulating climacteric intensity in melon through QTL stacking

**DOI:** 10.1093/hr/uhac131

**Published:** 2022-06-03

**Authors:** Miguel Santo Domingo, Lorena Areco, Carlos Mayobre, Laura Valverde, Ana Montserrat Martín-Hernández, Marta Pujol, Jordi Garcia-Mas

**Affiliations:** Centre for Research in Agricultural Genomics (CRAG) CSIC-IRTA-UAB-UB, Edifici CRAG, Campus UAB, Bellaterra, 08193 Barcelona, Spain; Centre for Research in Agricultural Genomics (CRAG) CSIC-IRTA-UAB-UB, Edifici CRAG, Campus UAB, Bellaterra, 08193 Barcelona, Spain; Centre for Research in Agricultural Genomics (CRAG) CSIC-IRTA-UAB-UB, Edifici CRAG, Campus UAB, Bellaterra, 08193 Barcelona, Spain; Centre for Research in Agricultural Genomics (CRAG) CSIC-IRTA-UAB-UB, Edifici CRAG, Campus UAB, Bellaterra, 08193 Barcelona, Spain; Centre for Research in Agricultural Genomics (CRAG) CSIC-IRTA-UAB-UB, Edifici CRAG, Campus UAB, Bellaterra, 08193 Barcelona, Spain; Institut de Recerca i Tecnologia Agoralimentàries (IRTA), Edifici CRAG, Campus UAB, Bellaterra, 08193 Barcelona, Spain; Centre for Research in Agricultural Genomics (CRAG) CSIC-IRTA-UAB-UB, Edifici CRAG, Campus UAB, Bellaterra, 08193 Barcelona, Spain; Institut de Recerca i Tecnologia Agoralimentàries (IRTA), Edifici CRAG, Campus UAB, Bellaterra, 08193 Barcelona, Spain; Centre for Research in Agricultural Genomics (CRAG) CSIC-IRTA-UAB-UB, Edifici CRAG, Campus UAB, Bellaterra, 08193 Barcelona, Spain; Institut de Recerca i Tecnologia Agoralimentàries (IRTA), Edifici CRAG, Campus UAB, Bellaterra, 08193 Barcelona, Spain

## Abstract

Fruit ripening is one of the main processes affecting fruit quality and shelf life. In melon there are both climacteric and non-climacteric genotypes, making it a suitable species to study fruit ripening. In the current study, in order to fine tune ripening, we have pyramided three climacteric QTLs in the non-climacteric genotype “Piel de Sapo”: *ETHQB3.5*, *ETHQV6.3* and *ETHQV8.1*. The results showed that the three QTLs interact epistatically, affecting ethylene production and ripening-related traits such as aroma profile. Each individual QTL has a specific role in the ethylene production profile. *ETHQB3.5* accelerates the ethylene peak, *ETHQV6.3* advances the ethylene production and *ETHQV8.1* enhances the effect of the other two QTLs. Regarding aroma, the three QTLs independently activated the production of esters changing the aroma profile of the fruits, with no significant effects in fruit firmness, soluble solid content and fruit size. Understanding the interaction and the effect of different ripening QTLs offers a powerful knowledge for candidate gene identification as well as for melon breeding programs, where fruit ripening is one of the main objectives.

## Introduction

Fruit development is one of the most important and energy-consuming processes during plant development. Ripening is the last step of this process, producing several biochemical and physiological changes in the fruit and making it attractive for animal and human consumption. Moreover, ripening has a significant role in plant breeding as it has a major effect in fruit shelf life and fruit quality. During this process, fruits undergo several changes, such as softening due to cell wall degradation or accumulation of pigments [1–3]. These changes happen in both climacteric and non-climacteric fruits. The difference between both types is the presence of a peak in ethylene production coupled to an increase in the respiration rate in the climacteric ones, or its absence in the non-climacteric [4]. The classical model organism to study fruit ripening has generally been tomato, due to the availability of several mutants with delayed or failed climacteric ripening [3]; but, as tomato is a climacteric fruit, there is much less information about non-climacteric ripening.

Melon (*Cucumis melo* L.) has emerged as an interesting model to study fruit ripening, as it has both climacteric and non-climacteric varieties within the same species [5]. In melon fruits, some ripening traits are dependent on ethylene, as aroma production or abscission layer formation, and other traits are independent, as carotenoid synthesis, sugar accumulation or, partially, flesh softening [6]. Also, the availability of different mapping populations, such as Recombinant Inbred Lines (RILs) [7, 8] or Introgression Lines (ILs) [9, 10], together with sequenced genome [11], makes melon a suitable model for studying both climacteric and non-climacteric fruit ripening.

The availability of IL populations makes possible to mendelize and study QTLs minimizing the effect of the genetic background. These populations have been used as genetic resources for several crops, as tomato [12], strawberry [13] or peach [14]. In melon, they have been widely used to study different traits, including ripening [9, 10, 15, 16]. In previous studies, two IL populations were used to dissect climacteric ripening in a non-climacteric background. In an IL population with non-climacteric genetic background of Piel de Sapo (PS) and the non-climacteric Songwan Charmi (SC) as donor parent, two QTLs were found triggering a climacteric response [17, 18]. Located in chromosome 3 and 6, they were named *ETHQB3.5* and *ETHQV6.3*, respectively. Besides, in an IL population with the same recurrent parent PS as genetic background, and Védrantais (Ved, climacteric) as donor parent, another major QTL was found, located in chromosome 8 and named *ETHQV8.1* [19].

The effect of these QTLs has been previously studied. *ETHQB3.5* was found to provoke the production of a peak of ethylene, compared to PS and SC [17, 18]. *ETHQB3.5* is located within the interval 26 000 631 – 28 759 416 bp in chromosome 3 (melon genome v3.6.1) [20], but the causal gene is still unknown. *ETHQV6.3*, encoded by a NAC transcription factor (MELO3C016540.2), is also capable of producing a climacteric response in PS, and increase and advance the ethylene production when both *ETHQB3.5* and *ETHQV6.3* alleles from SC are present in PS [18]. In a climacteric background, MELO3C016540.2 mutants showed a delayed production of ethylene [21]. *ETHQV8.1*, located in the interval 9 603 217 – 9 757 373 bp in chromosome 8 (melon genome v3.6.1), has been also studied in both climacteric and non-climacteric genetic backgrounds. In a non-climacteric background, introgressing the climacteric allele produces a climacteric response, and in a climacteric background, introgressing the non-climacteric allele produces a delay and a decrease in ethylene production [19]. However, we do not know the relationship between these three QTLs when they are combined in the same line and the consequences for melon ripening process and fruit quality.

The aim of this work is to study the effect of three QTLs, *ETHQB3.5, ETHQV6.3* and *ETHQV8.1*, in the non-climacteric background of PS and their possible interactions, to further modulate the climacteric response and associated traits happening during melon fruit ripening.

## Results

### Fruit ripening behavior and fruit quality of the parental lines

**Figure 1:**
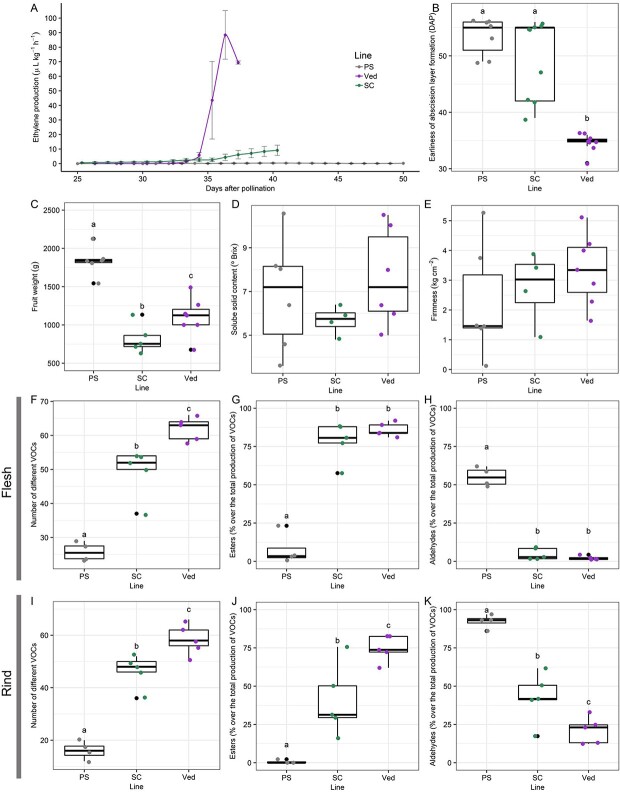
Phenotypic evaluation of the three parental lines PS, SC and Ved used in the study, showing: A) ethylene production (ETH), B) earliness of abscission layer formation (EALF), C) fruit weight (FW), D) soluble solid content (SSC), E) flesh firmness (FIR), F) diversity of VOCs in flesh, G) percentage of esters over the total amount of VOCs in flesh, H) percentage of aldehydes over the total amount of VOCs in flesh, I) diversity of VOCs in rind, J) percentage of esters over the total amount of VOCs in rind, K) percentage of aldehydes over the total amount of VOCs in rind. Letters represent significant difference between groups (p-value <0.05).

The three parental lines used in this study had different fruit ripening behavior (Figure 1). Piel de Sapo (PS) is a Spanish variety, belonging to *melo* subspecies and *inodorous* group. It produced a low amount of ethylene, insufficient to trigger the climacteric response. We considered PS as a classical non-climacteric cultivar [19]. Songwan Charmi (SC) is a Korean accession belonging to the *agrestis* subspecies, considered non-climacteric, but presenting some phenotypes associated with the climacteric response, as the production of aroma. It also produced ethylene during ripening, although in much less quantity if compared to climacteric types. Védrantais (Ved) is a French variety, from the *melo* subspecies as PS, and the *cantaloupensis* group. It had a typical climacteric fruit ripening behavior, with a sharp ethylene peak and noticeable related climacteric traits as abscission layer formation at 35 days after pollination (DAP) (Figure 1A, B).

Regarding fruit quality phenotypes, PS produced significantly bigger fruits compared to SC and Ved. Also, SC had the smallest fruits of the three parental lines (Figure 1C). We did not observe significant differences among the three parental lines in soluble solid content nor firmness of the flesh (Figure 1D, E) (Table S1).

When we studied the aroma profile, the three lines behaved differently. In flesh tissue, Ved accumulated the biggest amount of total volatile organic compounds (VOCs) followed by SC, and PS was the one accumulating the least amount of VOCs, being the differences significant (p < 0.05) among the three genotypes (Table S2A). In rind tissue, both PS and SC accumulated low amount of VOCs, and Ved significantly (p < 0.05) accumulated more VOCs than the other two genotypes (Table S2B). Looking at the number of different compounds produced by each line, Ved was also producing the most diverse aroma profile in both tissues, with around 60 different VOCs, followed by SC with 50 and PS with less than 30 different VOCs in flesh and less than 20 different compounds in rind tissue (Figure 1F, I) (Table S2). PS had a non-climacteric aroma profile, being aldehydes the major component in both rind and flesh tissues (92% and 55% of total amount of volatile compounds, respectively) with a lesser accumulation of esters (Figure 1G, H, J, K) (Table S3). Ved had a major component of esters in both rind and flesh (75.5% and 86%, respectively) and a minor component of aldehydes, although the total production of aldehydes was similar to PS in flesh and significantly bigger in rind (p < 0.05) (Figure 1G, H, J, K) (Table S3). In the case of SC, it had a different VOCs profile depending on the tissue. Regarding flesh tissue, although the total amount of produced VOCs is half the one found in Ved, it had a similar profile with a major component of esters (78%) (Figure 1G, H) (Table S3A, B). When focusing into the rind tissue, the production of VOCs was also reduced compared to Ved, and the profile also changed, with an intermediate behavior between Ved and PS with a mean of 40% of VOCs being esters and 40% aldehydes (Figure 1J, K) (Table S3C, D). Regarding other minor compounds, SC was the only line producing alkanes and furans in the rind, while PS produced furans in the flesh and phenols in rind (Table S3).

In a deeper analysis, when looking into the compounds that are most related to melon aroma such as (E, Z)-2,6-Nonadienal, Nonanal, (Z)-6-Nonenal, Ethyl acetate, Ethyl 2-methylbutanoate and Ethyl butanoate (Table S4A) [22], although SC is more similar to Ved than to PS regarding aroma profile, it produces more melon and cucumber-like aromas, while Ved produces more floral and fruity ones (Table S4B, C). Otherwise, PS has a general low production of these VOCs, both in flesh and rind tissues (Table S4B, C).

### Pyramiding ILs with 2 and 3 QTLs

**Figure 2:**
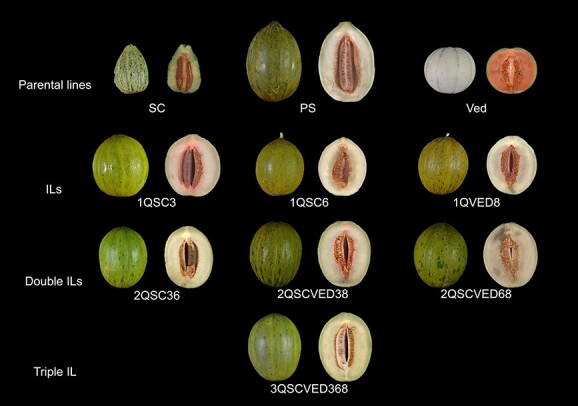
External and internal visualization of the fruits of the lines used in this study, including the parental lines. IL = Introgression Line, SC = Songwan Charmi, PS = Piel de Sapo, Ved = Védrantais.

We developed a collection of ILs in the PS background with homozygous introgressions covering one, two or three QTLs related with climacteric ripening, coming from two different parental lines (SC for *ETHQB3.5* and *ETHQV6.3*, and Ved for *ETHQV8.1*) (Figure 2) in two generations. The ILs developed in previous studies were renamed consistently with the new developed ILs (Table 1).

**Table 1:** Melon lines used in this work. SC = Songwan Charmi, VED = Védrantais

**Melon line** ^**a**^ **name**	**QTL (allele-code)**	**Previous line name**	**Reference**
1QSC3	SC-*ETHQB3.5*	8 M35	Moreno *et al*. 2008 [17] Vegas *et al*. 2013 [18]
1QSC6	SC- *ETHQV6.3*	8 M40	Vegas *et al*. 2013 [18]
1QVED8	VED-*ETHQV8.1*	VED8.2	Pereira *et al*. 2020 [19]
2QSC36	SC-*ETHQB3.5* SC- *ETHQV6.3*	8 M31	Vegas *et al*. 2013 [18]
2QSCVED38	SC-*ETHQB3.5* VED-*ETHQV8.1*	-	This work
2QSCVED68	SC- *ETHQV6.3* VED-*ETHQV8.1*	-	This work
3QSCVED368	SC-*ETHQB3.5* SC- *ETHQV6.3* VED-*ETHQV8.1*	-	This work

a The genetic background of all the lines is Piel de Sapo (PS)

### Fruit ripening behavior of the developed ILs

The main goal of this study was to dissect the ripening behavior of the developed ILs to understand the role of each QTL and their interactions.

**Figure 3:**
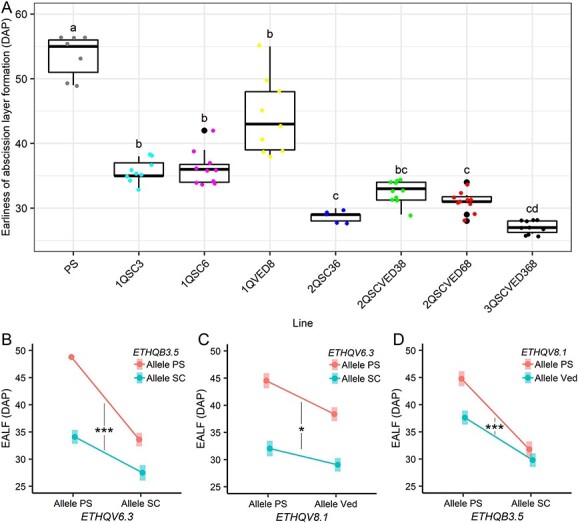
A) Representation of the phenotype Earliness of abscission layer formation (EALF) in the ILs. Significance based on t-test p-value <0.05. B), C), and D) Interaction plots showing the two-by-two interactions of the three studied QTLs. Significance is calculated through a multiple linear model.

Regarding the climacteric symptoms earliness of aroma production (EARO), earliness of abscission layer formation (EALF) and harvest date (HAR), a Principal Component Analysis showed that only one component explained 98% of the variation (Figure S2). EALF is the trait which covariates more with this component, so, we analyzed this trait as representative of the three main climacteric symptoms. After model selection, we selected the multiple linear model with the single effects and the three double interactions as the best fitting model (R^2^ = 0.86). Taking as reference PS, with the non-climacteric alleles for the three QTLs, the linear model showed a significant predicted advance in the symptoms for *ETHQB3.5*, *ETHQV6.3* and *ETHQV8.1* (Figure 3) (Table 2). Also, all the double interactions were predicted as significant (Figure 3) (Table 2). As seen in the model, the three double interaction effects are significantly “less than additive”, noticed in the different signs of the three main effects compared to the three interaction effects. The model predicts a minor advance in EALF when introgressing more than one QTL than the expected by the addition of the main effects of these QTLs (Table 2) (Figure S3).

**Table 2:** Multiple linear model used to analyze the earliness of abscission layer formation (EALF) in the ILs. R^2^ = 0.86

**Coefficients:**	**Effect**	**Estimate**	**Std. Error**	**t value**	**Pr(>|t|)**	**Significance**
PS	Intercept	53.16	0.97	54.715	< 2e-16	***
*ETHQB3.5-SC*	Main	−17.29	1.20	−14.397	< 2e-16	***
*ETHQV6.3-SC*	Main	−16.78	1.18	−14.17	< 2e-16	***
*ETHQV8.1-VED*	Main	−8.73	1.20	−7.272	6.19E-10	***
*ETHQV6.3-SC*:*ETHQV8.1-VED*	Interaction	3.16	1.35	2.343	0.022226	*
*ETHQB3.5-SC*:*ETHQV8.1-VED*	Interaction	5.17	1.35	3.835	0.000289	***
*ETHQB3.5-SC*:*ETHQV6.3-SC*	Interaction	8.59	1.34	6.415	1.95E-08	***

Regarding the semi-quantitative ripening-related level of abscission (ABS), it shows a statistically significant interaction between *ETHQB3.5* and *ETHQV6.3* (p-value = 0.005). While in PS the abscission level is low, when introgressing one of the QTLs coming from SC most of the melons presented complete abscission from the plant. On the other hand, when introgressing the Ved allele from *ETHQV8.1* the level of abscission increased but not as much as for SC alleles in chromosome 3 or 6 (Figure S4).

**Figure 4:**
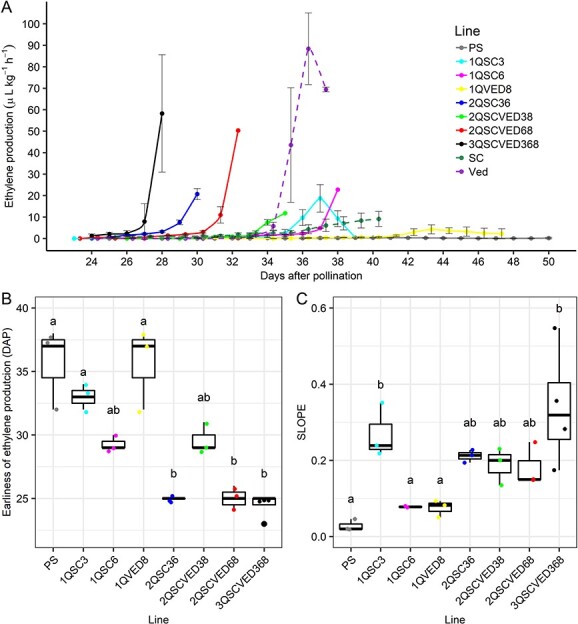
A) Ethylene production (ETH) of the ILs during fruit ripening, and in dashed lines, the two donor parents Ved and SC. B) earliness of ethylene production (DAPE) and C) slope of the log10 transformed data in the exponential part of the peak (SLOPE). Significance fixed at p-value <0.05.

The ethylene production of each of the ILs developed is presented in Figure 4A. With the single introgression of any of the QTLs, PS turned climacteric, but in different degrees. While *ETHQB3.5* and *ETHQV6.3* provoked an important peak of ethylene production around 36–37 DAP, *ETHQV8.1* only triggered a flat peak of ethylene production around 43–45 DAP. In the double ILs, we observed a diverse degree of climacteric, from an early one in 2QSC36 to a late one in 2QSCVED38. Also, we can observe that when pyramiding more than one QTL, an earlier climacteric response and a higher production of ethylene was induced. With the triple IL 3QSCVED368 we observed the sum of both effects, earlier and higher production, being the line with the highest climacteric behavior. While Ved had its ethylene production peak at 36 DAP (Figure 1), 3QSCVED368 had it at 28 DAP, one week earlier.

When we analyzed ethylene production related traits, we saw that for earliness of ethylene production and the peak of this production, a significant effect of *ETHQV6.3* was detected, and the interaction between the two QTLs *ETHQV8.1* and *ETHQV6.3* and the triple one was also significant (Figure 4B, Table S5A, B). Concerning the SLOPE, which is related with the sharpness of the peak explaining how quick the ethylene production increases, the model predicted a significant effect of *ETHQB3.5* narrowing the peak, and also an interaction between *ETHQV6.3* and *ETHQV8.1* when they were together (Figure 4C, Table S5C). For ethylene production related traits, our data suggest that *ETHQV6.3* has a significant effect for the earliness of the peak, and *ETHQB3.5* for its sharpness. On the other hand, although *ETHQV8.1* does not have a significant effect on its own, it enhanced the effect of the other two QTLs.

### Aroma production and fruit quality of the developed ILs

**Figure 5:**
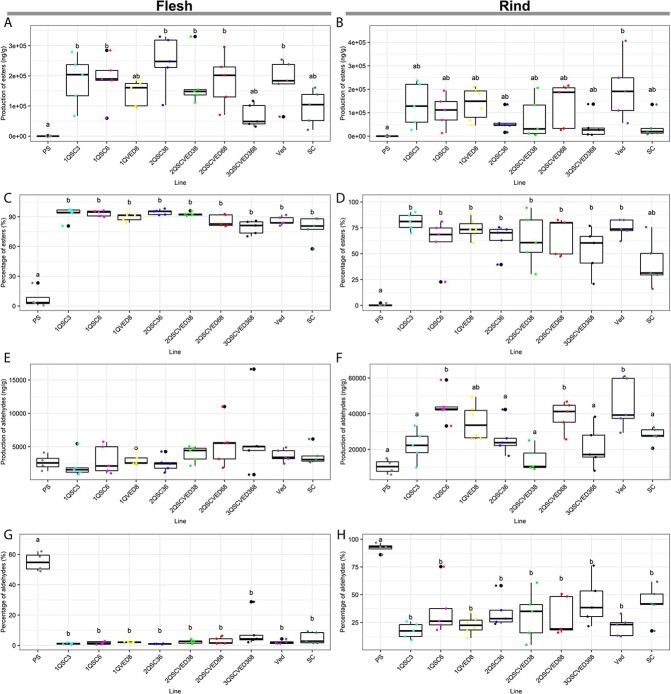
Production of esters and aldehydes, showing both total relative production respect to the internal standard (3-hexanone) and percentage over the total VOCs. A), C), E) and G) in flesh tissue, and B), D), F) and H) in rind tissue.

Another important trait for fruit quality in melon is the volatile compounds composition, as their combination shapes the aroma profile of the fruit. The introgression of the climacteric ripening QTLs produced a significant increase in the total amount of volatiles production, compared to PS, in both fruit flesh and rind (Figure 5) (Table S2A, B) Analyzing the different compounds, we can observe that, although there was variability within each line, the most important effect was due to the accumulation of esters (mainly acetic acid esters) in fruit flesh and rind, and the accumulation of aldehydes (mainly benzaldehyde) in the rind (Figure S5). In PS, there was little production of esters in both rind and flesh, but when we introgressed the QTLs, the fruits significantly accumulated a bigger quantity of esters in flesh, even reaching the levels of esters production in Ved (Table S3A) (Figure 5A). There was also an increase of ester production in rind, although generally, the levels reached were not higher than Ved (Table S3C) (Figure 5B). In both rind and flesh, the accumulation of esters became the main component of the aroma profile in the introgression lines (more than 75% and 50% of total volatiles in flesh and rind, respectively), similar to the climacteric cultivar Ved and significantly higher than in PS in all the pyramided lines (Table S3B, D) (Figure 5C, D). Regarding the aldehydes (the second group in importance in melon fruit aroma profile), the total amount was very variable. In flesh, there was not a significant increase in any of the lines, even in the other parents Ved and SC (Table S3A) (Figure 5E). In rind tissue, although we did observe a significant increase in some lines, the behavior was very variable without a clear trend (Table S3C, Figure 5F). However, looking at the percentage of aldehydes we did observe a significant trend. While in PS the aldehydes were the main contributor to the aroma profile in both rind and flesh (more than 90% and 50% respectively), in the introgression lines the percentage was significantly lower in both tissues probably due to the high percentage of esters (Table S3B, D) (Figure 5G, H). If we look at the other analyzed compounds, we can observe that when we introgressed the climacteric QTLs, the lines lost the capacity of accumulating furans in fruit flesh (Table S3A). We can also observe a bigger accumulation of alcohols when introgressing *ETHQV8.*1, approaching the levels of SC and Ved in flesh and rind (Table S3A, C). Also, when introgressing the QTLs, the fruits produced more terpenes, especially the lines with *ETHQB3.5*, but not reaching the levels of Ved (Table S3A, C).

Moreover, we studied the association of all the VOCs with individual QTLs, considering the large variability within genotypes. Using linear models, we selected the additive model to see the individual association of each single QTL with the compounds, and then selected the models which explained more than 40% of the variance (adjusted R^2^ > 0.4), indicating a major genetic control of the trait by the QTLs individually. In the case of rind, two compounds met this condition: eucalyptol and benzeneacetaldehyde (Table S6). Both were associated significantly with *ETHQB3.5,* and highly associated with *ETHQV6.3* in the case of benzenacetaldehyde, and with *ETHQV8.1* in the case of eucalyptol (Table S6A, B). When focusing on the flesh, four compounds were associated with the QTLs. Eucalyptol appeared also associated with *ETHQV8.1* and *ETHQB3.5* in flesh, and phenylethyl alcohol with *ETHQV8.1* and *ETHQV6.3* (Table S6C, D). Moreover, another compound, 1-octen-3-ol was highly associated with *ETHQV8.1* (Table S6F). All of these compounds increased their production with the donor parent allele. Interestingly, the last compound highly associated with one QTL was propanoic acid, 2-methyl-, ethyl ester, one of the compounds described as main contributor of melon aroma (Table S4A). We detected a highly significant association (p < 0.01) of this compound with *ETHQV8.1*, increasing its production when introgressing the donor allele (Table S6E).

Regarding the analysis of the main contributors to the melon aroma (Table S4A), we can observe different behaviors. Although SC produced the cucumber-like aroma compound 2–6 nonadienal in rind, none of the ILs recovered this phenotype, while we observed non-significant increase of its production in flesh in most of the ILs (Table S2A). Regarding the melon-like aroma compounds nonanal and 6-nonenal, we detected a significant increase of nonanal in flesh in the line 3QSCVED368 (p < 0.05), and non-significant increase in other introgression lines, such as 1QSC6 and 2QSC36, when compared to PS (Table S2A). The major effect was observed in the floral and fruity aroma compounds. By introgressing the QTLs, we could mostly recover the phenotype of Ved in both rind and flesh for those compounds. Individually, there was an increase in the production of ethyl acetate, and butanoic acid ethyl ester in 1QVED8, producing bigger amounts than Ved, but we did not observe this trend in other lines carrying *ETHQV8.1*. In these compounds, the lesser effect was observed when introgressing *ETHQB3.5*, being 1QSC3, 2QSC36, 2QSCVED38 and 3QSCVED368 similar to PS (Table S2A, B). For the other compounds, although there was an increase in the ILs compared to PS, we did not observe significant changes or trends (Table S2).

For the fruit quality traits, there was no significant effect of either of the QTLs, and we did not observe significant differences among the lines compared to PS for fruit weight, sugar content or firmness (Figure S6). However, we detected a trend of smaller fruits for any of the introgressed QTLs (Figure S6A).

## Discussion

In this study, we present the pyramiding of three QTLs involved in climacteric ripening coming from two different populations sharing the non-climacteric recurrent parental line PS. While *ETHQB3.5* and *ETHQV6.1* come from an IL population with the non-climacteric donor parent Songwan Charmi PI 161375 [9, 23], *ETHQV8.1* comes from an IL population with the climacteric Ved as the donor parent [19]. This pyramiding strategy has been used in different crops and with several traits, such as improving yield under stress conditions or blast resistance in rice [24, 25], fungi resistance in pea [26] or insect resistance in soybean [27]. Similar strategies, but with a single donor parent, have been used to improve yield in different crops, as cucumber [28] or tomato [29]. In the case of melon, there have been studies of pyramided QTLs to improve resistance to gummy stem blight [30] and to advance ripening [18]. In all of these studies, the use of ILs with combinations of different QTLs has proved as a powerful tool to uncover their effect in diverse phenotypes, as well as their interactions. However, pyramiding genes involved in fruit quality or ripening has not been yet fully implemented in cultivated crops [31]. With the genomic tools available in melon, such as the reference genome and more than one thousand accessions resequenced [11, 32], molecular markers can be easily obtained and used for shortening the process of pyramiding QTLs by MAS.

### QTLs interact to modulate the climacteric ripening behavior in melon fruits

To date, most of the work about climacteric ripening has been done in tomato fruits. Several mutants with delayed ripening have been used to characterize genes controlling climacteric response [3, 33, 34]. However, inducing early climacteric ripening in a non-climacteric background, as we present in this work, has not been addressed.

Although the effects of the three QTLs here described have been studied separately, this is the first time that they are combined and analyzed in the same experiment. The three QTLs produce a climacteric response in PS independently, with production of ethylene and related climacteric symptoms. *ETHQV8.1* was the weakest QTL, provoking a lower and delayed ethylene peak (Figure 4A). *ETHQB3.*5 caused a fast production of the ethylene peak (Figure 4C), and *ETHQV6.3* significantly advanced both the ethylene production and the peak intensity (Figure 4B). When observing the climacteric symptoms, the analysis showed a significant effect when introgressing *ETHQV6.3*, *ETHQB3.5* or *ETHQV8.1* (Table 2), being the effect bigger for the first two QTLs. This bigger effect of the QTLs coming from SC was previously observed [18, 19] and *ETQHQV8.1* has been reported to be unstable, provoking a mild climacteric behavior depending on the environmental conditions [19]. When combining two of the QTLs in the same line, we observed a statistical interaction in the three combinations, bringing out a potential interaction among the causal genes. The general trend of these QTLs is a “less than additive” effect, being this model significant for the three double interactions, predicting a lighter effect when introgressing two QTLs that the expected if it was additive (Table 2) (Figure S3). As melons are reported to start ripening at around 30 DAP, coinciding with the starting of sucrose accumulation [35], maybe the mathematically estimated additive value is too early, during the fruit growth stage. Melons carrying two or three of the QTLs should ripe before 20 DAP when the fruit is still under development and probably it is not ready for ripening. Thus, linear models seem an appropriate tool for this type of experiment, as they can detect statistical interactions among variables, in this case QTLs, and have been used previously for this kind of studies [26, 29]. After model selection, the most suitable model was the one with the three double interactions that explains 88.55% of the variance, evidencing also that there is no need to include the triple interaction to predict the ripening behavior of the population. This significance of all the interactions may be indicating that the phenotype is due to the interactions between the different alleles of those QTLs.

When we looked individually to each of the QTLs, we observed a clear effect of them in the ethylene production profile. *ETHQV6.3* advanced the ethylene production, as well as the ethylene peak. This result matches previous studies showing that TILLING mutants of MELO3C016540.2 in a climacteric background delay the production of ethylene but not the amount or the shape of the peak [21]. In the case of *ETHQB3.5* and *ETHQV8.1*, although the causal genes are not yet known, our results could help in their identification. Our results suggest that the role of *ETHQB3.5* is to narrow the ethylene peak, decreasing the days from the start of the ethylene production to the maximum production. In a recent study, two candidate genes have been suggested in the *ETHQB3.5* interval: MELO3C011432.2, a WRKY family transcription factor, and MELO3C011365.2, related with signal transduction [36]. *ETHQV8.1*, although it has a weaker effect compared to the other two QTLs, has an important role in enhancing the response in combination with both of them. Three candidate genes have been suggested in the region of this QTL: MELO3C024516.2, MELO3C024518.2 and MELO3C024520.2, encoding a demethylase, a negative regulator of ethylene signal transduction and an ethylene responsive transcription factor, respectively [19]. All three of them may play a role in fruit ripening, and our work can contribute to understand the specific function of *ETHQV8.1*.

Our results suggested an important role of the genetic background, since *ETHQB3.5* and *ETHQV6.3,* both carrying SC alleles, behave differently when introgressed in PS. This effect reveals the complex architecture of ripening in melon, as it has been reported before in other crops [31]. Moreover, when introgressing two (2QSCVED68) or three (3QSCVED368) QTLs in PS, we can obtain a line that is even more climacteric than Ved, which is known to be a typical climacteric cantaloupe type. This finding suggests that there are some key genes governing the climacteric response, and combining their natural variation, we can convert a non-climacteric variety with long shelf life into an early maturation and aromatic melon line.

By combining these three QTLs we can obtain melons with different degrees of climacteric response. As ethylene production is one of the main traits affecting fruit shelf life, here we have implemented a tool to control its production with only three major QTLs that can potentially be fine-tuned using other minor QTLs found in previous studies [19]. This approach could be used to develop new melon cultivars with different degrees of shelf life in both directions. We could develop aromatic climacteric varieties from a non-climacteric variety as shown in this study; and from a climacteric cultivar as Ved, pyramiding ILs with delayed ripening [10, 16], we could develop long shelf life climacteric varieties.

### The aroma profile can be modified in an *inodorus* melon

Piel de Sapo (PS), the recurrent parent of the ILs, belongs to the *inodorous* group, the term meaning “no aroma” in Latin. As a non-climacteric variety, PS is characterized by a mild or even undetectable aroma profile. In this study, we introgressed different QTLs in the PS background triggering a climacteric response during ripening, and all of them induced accumulation of ester compounds and a meaningful change in the aroma profile of the fruit. Regarding the aroma profile, we cannot differentiate the pyramided lines when smelling them, as all of them are climacteric and produce a similar aroma. The analysis of melon fruits by GC–MS showed that for the climacteric lines, the main contributor to the aroma profile are esters [22, 37], while the non-climacteric PS does not produce esters, being aldehydes the main contributors to the aroma profile. The line 3QSCVED368, the earliest climacteric line, had a lower production of esters than the other lines, suggesting that this effect may be due to its quick ripening and fruit abscission, preventing the accumulation of esters. Alcohol acyl-transferases, the enzymes driving esters biosynthesis, have been reported to be expressed later than 30 DAP in melon [38], later than the harvest date of 3QSCVED368, probably leading to a minor accumulation of esters in this line compared to the other ILs. We also detected changes in the composition of the aroma in the pyramided lines. The introgression of *ETHQV8.1* from Ved enhances the production of fruity and floral aromas, and the introgression of *ETHQB3.5* does not produce a strong effect in the main contributors to melon aroma (Table S4). Therefore, even if *ETHQV8.1* has not the highest impact in ethylene production, it seems to affect key VOCs implicated in melon aroma.

Our results suggest a potential use of different QTLs to shape the aroma profile in melon. Although we observed a high variation regarding VOCs accumulation in both rind and flesh tissues, some compounds seemed to be associated with a particular QTL, making it possible to fine-tune the melon aroma by pyramiding different QTLs. Moreover, we demonstrated that is possible to introduce aroma in an *inodorous* cultivar by introgressing any of the three QTLs, suggesting that the control of aroma production depends mainly on ethylene production.

### Minor effect in fruit quality caused by the introgressions

Our results showed that there were not significant differences between PS and the pyramided lines related to fruit quality traits as weight, soluble solid content and firmness. However, there was a trend showing a decrease in fruit weight in all the ILs. A relationship between ripening and fruit size has been reported in tomato [31] and melon [39]. However, the ILs here reported contain large regions with many genes, making it possible that the effect on fruit size may be the result of other genes present inside the introgression. In the case of fruit firmness, although the softening of the fruit is partially regulated by ethylene [6], we did not detect any significant effect.

The results of this study demonstrate the possibility of pyramiding different QTLs in the same genetic background to shape and fine-tune the ripening process in melon fruits. With only three QTLs, we can obtain a line with an extreme climacteric behavior in the non-climacteric cultivar Piel de Sapo background. In addition, we can modify the aroma profile of the ripe fruit using these QTLs. The combination of these three major QTLs represents a powerful tool to modify and fine-tune climacteric fruit ripening in melon breeding programs.

## Materials and methods

### Plant material

The starting lines derive from two different IL populations, both constructed in the genetic background of the Piel de Sapo (PS) variety (*inodorous* group). Three of the initial lines belong to a previously characterized IL population [9, 18] with the Korean accession Songwan Charmi (SC, PI 161375) as the donor parent. These three lines are 1QSC3 (previously known as 8 M35), 1QSC6 (previously named 8 M40) and 2QSC36 (known as 8 M31), carrying the QTLs *ETHQB3.5*, *ETHQV6.3* and both, respectively. The second IL population used in this study has the variety Védrantais (Ved, *cantalupensis*) as the donor parent, and is still under construction. The line 1QVED8 was previously described as VED8.2, carrying the QTL *ETHQV8.1* [19]. For a more understandable and consistent naming, the initial lines were renamed. All lines are described in Table 1.

### Breeding scheme

The pyramided ILs with double and triple introgressions were developed by crosses and Marker Assisted Selection (Figure S1). To construct the double ILs 2QSCVED38 and 2QSCVED68, two single ILs were first crossed and then self-pollinated. 192 F2 plants of each cross were genotyped with flanking markers selecting two individual plants containing the two introgressions in homozygosis, which were self-pollinated to obtain the progeny for phenotyping. For the triple IL, the already developed double introgression line 2QSC36 was crossed with IL 1QVED8, and then self-pollinated. 384 F2 individuals were genotyped and two plants carrying the three QTLs in homozygosis were selected, which were self-pollinated to obtain the progeny for phenotyping. With this scheme, 7 lines with all the possible homozygous combinations of the three QTLs were developed in two generations, and another generation was needed to multiply the seeds.

**Table 3:** Traits evaluated in the experiment

**Category**	**Trait (units)**	**Code**
Fruit quality	Fruit weight (g)	FW
	Soluble solid content (°Brix)	SSC
	Flesh firmness (kg cm^−2^)	FIR
Fruit ripening	Earliness of aroma production (DAP^*^)	EARO
	Aroma production	ARO
	Earliness of abscission layer formation (DAP^*^)	EALF
	Level of abscission	ABS
	Harvest date (DAP^*^)	HAR
Ethylene production	Ethylene production (μL kg^−1^ h^−1^)	ETH
	Earliness of ethylene production (DAP^*^)	DAPE
	Earliness of the ethylene peak (DAP^*^)	DAPP
	Slope of the peak	SLOPE

a DAP Days after pollination

### DNA extraction and genotyping

DNA was extracted from young leaves with two different protocols depending on the use of the DNA: for quick genotyping and short-term storage, alkaline-lysis extraction was used [40], and for genotyping and long-term storage, a CTAB protocol [41] with some modifications was used [8].

The genotyping was performed using two SNP genotyping systems: KASPar SNP Genotyping System (KBiosciences, Herts, UK), and PACE2.0 SNP Genotyping System (3CR Bioscience, Essex, UK). Primers were designed following each genotyping system instructions (Table S7). Two of the QTLs were genotyped using flanking markers (*ETHQB3.5* and *ETHQV8.1*). *ETHQV6.3* was genotyped using a marker designed into the causal gene, MELO3C016540.2 [21].

### Growing conditions

Seedlings were germinated and grown in an indoor greenhouse (CRAG, Barcelona) under controlled conditions for three weeks. Growing of selected plants was performed in a greenhouse (Caldes de Montbui, Barcelona) in randomized blocks. In flowering time, pollinations were manually performed until one fruit per plant was obtained. For the development of the new lines, two generations were obtained in the same year during 2018 and 2019, and the phenotyping was performed during summer 2020. The harvest point was determined based on the presence of abscission layer, as this is a trait highly associated with ethylene production. Harvest date was fixed at: a) abscission date when the fruit abscised; b) five days after the appearance of the abscission layer; c) 56 days after pollination (DAP) when fruits were non-climacteric and did not form abscission layer, considering them fully ripe at that point. For each genotype, ten plants were grown and depending on the fruit set of each genotype more than seven fruits were evaluated.

### Phenotyping

All traits related to fruit quality and ripening behavior are listed in Table 3. Traits were classified according to three different categories: fruit quality (fruit weight (FW), soluble solid content (SSC) and firmness (FIR)); fruit ripening-related (aroma production (ARO), earliness of aroma production (EARO), earliness of abscission layer formation (EALF), level of abscission (ABS) and harvest date (HAR)); and ethylene-related (maximum ethylene production (ETH), earliness of ethylene production (DAPE) defining the day when ethylene was detected, earliness of the ethylene peak (DAPP) and the slope of the peak measuring the log10 transformed data in the exponential part of the peak (SLOPE)). ARO and ABS were inspected daily from 20 days after pollination (DAP) until their appearance. ABS was recorded according to the following scale: 0 (absence of abscission layer); 1 (partial abscission layer formation); 2 (formation of an almost complete abscission layer and presence of a scar); and 3 (complete abscission layer formation and fruit abscission).

Fruit flesh firmness was recorded at harvest in different regions of the fruit (proximal, median and distal) with a penetrometer (Fruit Test™, Wagner Instruments), and the mean value was calculated. Flesh juice was used to measure total soluble solids content (Brix index) with a refractometer (Atago™). Fruit weight was measured at harvest.

### Ethylene production

Gas chromatography – mass spectrometry (GC–MS) was used to measure ethylene production of melon fruits *in planta* as previously described [42]. Briefly, melon fruits were covered with a sealed plastic bag, and then filled with atmospheric air. After 1 h, gas sample was extracted using a syringe and introduced in a vial for GC–MS analysis.

Ethylene production was monitored from 25 DAP until fruit harvest. For the ILs and Ved, ethylene was measured every other day while it was undetectable and every day when first detected. For the non-climacteric lines (PS and SC), we measured the atmosphere of the chamber every three days. In total, three to four fruits were analyzed per line. Data was expressed under the four ethylene-related traits: ETH, DAPE, DAPP and SLOPE (Table 3).

### Aroma profiling

The aroma profiling of the rind and the flesh tissue of melon fruits was analyzed at harvest with GC–MS as previously described [22]. In summary, 2 g of grinded frozen tissue were added to a vial, with 7 ml of saturated NaCl solution and 3-hexanone as internal standard. Until gas chromatograph (GC) mass spectrometer (MS) analysis was performed, tubes were stored up to one week at 4°C in the dark. Vials were analyzed with GC–MS using Solid Phase Micro Extraction (SPME) in a 7890A GC coupled to a 5975C MS and a GC PAL 80 autosampler (Agilent Technologies®, Santa Clara, CA). Volatile organic compounds (VOCs) were identified by comparing their mass spectra with both the NIST 11 library and their Kovats retention index. Comparison to the 3-hexanone internal standard peak area was used to estimate the relative content of each compound.

### Statistical analyses

Statistical analyses and graphical representations were performed using the software R v3.5.3 with the RStudio v1.0.143 interface [43].

Data should not contain any missing data in order to compute the analyses. Therefore, the harvest day value was imputed to non-climacteric fruits for EARO and EAFL when these traits were absent.

PCAs were performed with the R package “factoextra”. The multiple linear models were performed with the function “lm”, using as factor the correspondent allele of each QTL. Model selection was performed under Akaike Information Criterion. ANOVA and pairwise t-test were performed using R package “rstats” with Holm correction. In general, significance was fixed at p-value <0.05.

## Acknowledgements

This work was supported by grants AGL2015–64625-C2–1-R, RTI2018-097665-B-C2, SEV-2015-0533 and CEX2019-000902-S funded by Ministerio de Ciencia e Innovación MCIN/AEI/10.13039/501100011033 and FEDER, and by the CERCA Programme/Generalitat de Catalunya and 2017 SGR 1319 grant from the Generalitat de Catalunya to JGM. M.S.D. and L.V. were supported by grants BES-2017-079956 and PRE2018-086627 funded by MCIN/AEI/10.13039/501100011033 and by “ESF Investing in your future”. CM was supported by 2019 FI_B 00124 fellowship from the Secretaria d’Universitats i Recerca del Departament d’Empresa i Coneixement de la Generalitat de Catalunya and the co-funding of the European Social Fund (ESF “ESF is investing in your future”) from the European Union. Special thanks to Fuensanta García for assistance in field and lab operation and Martí Bernardo for assistance in data analyses.

## Author contributions

J.G.-M. and M.P. conceived and designed the research. M.S.D., LA, LV and CM performed the experiments. AMM-H contributed in the management of the plant material. M.S.D. analyzed the data and wrote the original draft. M.P. and J.G.-M. reviewed and edited the manuscript. All authors read and approved the manuscript.

## Data availability statement

The authors declare that the data supporting the study findings are presented in the article and additional supporting files are available from the corresponding authors (J.G.-M. and M.P.) upon request.

## Conflict of interest

The authors declare that they have no conflict of interest.

## Supplementary data

Supplementary data is available at *Horticulture Research * online.

## Supplementary Material

Web_Material_uhac131

## References

[1] Seymour GB, Manning K, Eriksson EM et al. Genetic identification and genomic organization of factors affecting fruit texture. J Exp Bot. 2002;53:2065–71. 12324530 10.1093/jxb/erf087

[2] Prasanna V, Prabha TN, Tharanathan RN. Fruit ripening phenomena-an overview. Crit Rev Food Sci Nutr. 2007;47:1–19. 17364693 10.1080/10408390600976841

[3] Giovannoni JJ . Fruit ripening mutants yield insights into ripening control. Curr Opin Plant Biol. 2007;10:283–9. 17442612 10.1016/j.pbi.2007.04.008

[4] Giovannoni JJ . Genetic regulation of fruit development and ripening. Plant Cell. 2004;16:S170–80. 15010516 10.1105/tpc.019158 PMC2643394

[5] Ezura H, Owino WO. Melon, an alternative model plant for elucidating fruit ripening. Plant Sci. 2008;175:121–9.

[6] Pech JC, Bouzayen M, Latché A. Climacteric fruit ripening: ethylene-dependent and independent regulation of ripening pathways in melon fruit. Plant Sci. 2008;175:114–20.

[7] Périn C, Hagen L, de Conto V et al. A reference map of Cucumis melo based on two recombinant inbred line populations. Theor Appl Genet. 2002;104:1017–34. 12582608 10.1007/s00122-002-0864-x

[8] Pereira L, Ruggieri V, Pérez S et al. QTL mapping of melon fruit quality traits using a high-density GBS-based genetic map. BMC Plant Biol. 2018;18:324. 30509167 10.1186/s12870-018-1537-5 PMC6278158

[9] Eduardo I, Arús P, Monforte AJ. Development of a genomic library of near isogenic lines (NILs) in melon (Cucumis melo L.) from the exotic accession PI161375. Theor Appl Genet. 2005;112:139–48. 16208502 10.1007/s00122-005-0116-y

[10] Pereira L, Santo Domingo M, Argyris J et al. A novel introgression line collection to unravel the genetics of climacteric ripening and fruit quality in melon. Sci Rep. 2021;11:11364. 34059766 10.1038/s41598-021-90783-6 PMC8166866

[11] Garcia-Mas J, Benjak A, Sanseverino W et al. The genome of melon (Cucumis melo L.). Proc Natl Acad Sci U S A. 2012;109:11872–7. 22753475 10.1073/pnas.1205415109 PMC3406823

[12] Bernacchi D, Beck-Bunn T, Emmatty D et al. Advanced backcross QTL analysis of tomato. II. Evaluation of near-isogenic lines carrying single-donor introgressions for desirable wild QTL-alleles derived from Lycopersicon hirsutum and L. pimpinellifolium. Theor Appl Genet. 1998;97:170–80.

[13] Urrutia M, Rambla JL, Alexiou KG et al. Genetic analysis of the wild strawberry (Fragaria vesca) volatile composition. Plant Physiol Biochem. 2017;121:99–117. 29100102 10.1016/j.plaphy.2017.10.015

[14] Serra O, Donoso JM, Picañol R et al. Marker-assisted introgression (MAI) of almond genes into the peach background: a fast method to mine and integrate novel variation from exotic sources in long intergeneration species. Tree Genet Genomes. 2016;12:96.

[15] Castro G, Perpiñá G, Monforte AJ et al. New melon introgression lines in a Piel de Sapo genetic background with desirable agronomical traits from dudaim melons. Euphytica. 2019;215:169.

[16] Perpiñá G, Cebolla-Cornejo J, Esteras C et al. ‘MAK-10’: a long shelf-life Charentais breeding line developed by introgression of a genomic region from Makuwa melon. HortScience. 2017;52:1633–8.

[17] Moreno E, Obando JM, Dos-Santos N et al. Candidate genes and QTLs for fruit ripening and softening in melon. Theor Appl Genet. 2008;116:589–602. 18094954 10.1007/s00122-007-0694-y

[18] Vegas J, Garcia-Mas J, Monforte AJ. Interaction between QTLs induces an advance in ethylene biosynthesis during melon fruit ripening. Theor Appl Genet. 2013;126:1531–44. 23443139 10.1007/s00122-013-2071-3

[19] Pereira L, Santo Domingo M, Ruggieri V et al. Genetic dissection of climacteric fruit ripening in a melon population segregating for ripening behavior. Hortic Res. 2020;7:187–18. 33328460 10.1038/s41438-020-00411-z PMC7603510

[20] Pereira L . Genetic dissection of fruit quality and ripening traits in melon. PhD thesis, Universitat Autònoma de Barcelona, Barcelona, 2018.

[21] Ríos P, Argyris J, Vegas J et al. ETHQV6.3 is involved in melon climacteric fruit ripening and is encoded by a NAC domain transcription factor. Plant J. 2017;91:671–83. 28493311 10.1111/tpj.13596

[22] Mayobre C, Pereira L, Eltahiri A et al. Genetic dissection of aroma biosynthesis in melon and its relationship with climacteric ripening. Food Chem. 2021;353:129484. 33812162 10.1016/j.foodchem.2021.129484

[23] Argyris JM, Pujol M, Martín-Hernández AM et al. Combined use of genetic and genomics resources to understand virus resistance and fruit quality traits in melon. Physiol Plant. 2015;155:4–11. 25594580 10.1111/ppl.12323

[24] Sandhu N, Dixit S, Swamy BPM et al. Marker assisted breeding to develop multiple stress tolerant varieties for flood and drought prone areas. Rice. 2019;12:8. 30778782 10.1186/s12284-019-0269-y PMC6379507

[25] Guan H, Hou X, Jiang Y et al. Feature of blast resistant near-isogenic lines using an elite maintainer line II-32B by marker-assisted selection. J Plant Pathol. 2019;101:491–501.

[26] Lavaud C, Baviere M, le Roy G et al. Single and multiple resistance QTL delay symptom appearance and slow down root colonization by Aphanomyces euteiches in pea near isogenic lines. BMC Plant Biol. 2016;16:166. 27465043 10.1186/s12870-016-0822-4 PMC4964060

[27] Ortega MA, All JN, Boerma HR et al. Pyramids of QTLs enhance host–plant resistance and Bt-mediated resistance to leaf-chewing insects in soybean. Theor Appl Genet. 2016;129:703–15. 26724806 10.1007/s00122-015-2658-y PMC4799260

[28] Robbins MD, Casler MD, Staub JE. Pyramiding QTL for multiple lateral branching in cucumber using inbred backcross lines. Mol Breeding. 2008;22:131–9.

[29] Gur A, Zamir D. Mendelizing all components of a pyramid of three yield QTL in tomato. Front Plant Sci. 2015;6:1096. 26697048 10.3389/fpls.2015.01096 PMC4678209

[30] Zhang N, Xu BH, Bi YF et al. Development of a muskmelon cultivar with improved resistance to gummy stem blight and desired agronomic traits using gene pyramiding. Czech J Genet Plant Breed. 2017;53:23–9.

[31] Wang R, Lammers M, Tikunov Y et al. The rin, nor and Cnr spontaneous mutations inhibit tomato fruit ripening in additive and epistatic manners. Plant Sci. 2020;294:110436. 32234221 10.1016/j.plantsci.2020.110436

[32] Zhao G, Lian Q, Zhang Z et al. A comprehensive genome variation map of melon identifies multiple domestication events and loci influencing agronomic traits. Nat Genet. 2019;51:1607–15. 31676864 10.1038/s41588-019-0522-8

[33] Vrebalov J, Ruezinsky D, Padmanabhan V et al. A MADS-box gene necessary for fruit ripening at the tomato ripening-inhibitor (Rin) locus. Science. 2002;296:343–6. 11951045 10.1126/science.1068181

[34] Manning K, Tör M, Poole M et al. A naturally occurring epigenetic mutation in a gene encoding an SBP-box transcription factor inhibits tomato fruit ripening. Nat Genet. 2006;38:948–52. 16832354 10.1038/ng1841

[35] Saladié M, Cañizares J, Phillips MA et al. Comparative transcriptional profiling analysis of developing melon (*Cucumis melo* L.) fruit from climacteric and non-climacteric varieties. BMC Genomics. 2015;16:440. 26054931 10.1186/s12864-015-1649-3 PMC4460886

[36] Oren E, Tzuri G, Dafna A et al. QTL mapping and genomic analyses of earliness and fruit ripening traits in a melon recombinant inbred lines population supported by de novo assembly of their parental genomes. Hortic Res. 2022;9:uhab081. 10.1093/hr/uhab081 PMC8968493 35043206

[37] Obando-Ulloa JM, Moreno E, García-Mas J et al. Climacteric or non-climacteric behavior in melon fruit: 1. Aroma volatiles. Postharvest Biol Technol. 2008;49:27–37.

[38] Yahyaoui FEL, Wongs-Aree C, Latché A et al. Molecular and biochemical characteristics of a gene encoding an alcohol acyl-transferase involved in the generation of aroma volatile esters during melon ripening. Eur J Biochem. 2002;269:2359–66. 11985619 10.1046/j.1432-1033.2002.02892.x

[39] Chatzopoulou F, Sanmartin M, Mellidou I et al. Silencing of ascorbate oxidase results in reduced growth, altered ascorbic acid levels and ripening pattern in melon fruit. Plant Physiol Biochem. 2020;156:291–303. 32987259 10.1016/j.plaphy.2020.08.040

[40] Lu J, Hou J, Ouyang Y et al. A direct PCR–based SNP marker–assisted selection system (D-MAS) for different crops. Mol Breeding. 2020;40:9.

[41] Doyle J . DNA Protocols for Plants. In: Hewitt GM, Johnston AWB, Young JPW, eds. Molecular Techniques in Taxonomy. Springer Berlin Heidelberg: Berlin, Heidelberg, 1991,283–93.

[42] Pereira L, Pujol M, Garcia-Mas J et al. Non-invasive quantification of ethylene in attached fruit headspace at 1 p.p.b. by gas chromatography–mass spectrometry. Plant J. 2017;91:172–83. 28370685 10.1111/tpj.13545

[43] R Core Team (2020). R: A Language and Environment for Statistical Computing. R Foundation for Statistical Computing, Vienna, Austria. https://www.gbif.org/tool/81287/r-a-language-and-environment-for-statistical-computing.

