# Supplementary figures and images for: Modulating climacteric intensity in melon through QTL stacking
Source: Hortic Res. 2022 Jun 3;9:uhac131. doi: 10.1093/hr/uhac131 (PMC9343914; doi:10.1093/hr/uhac131)

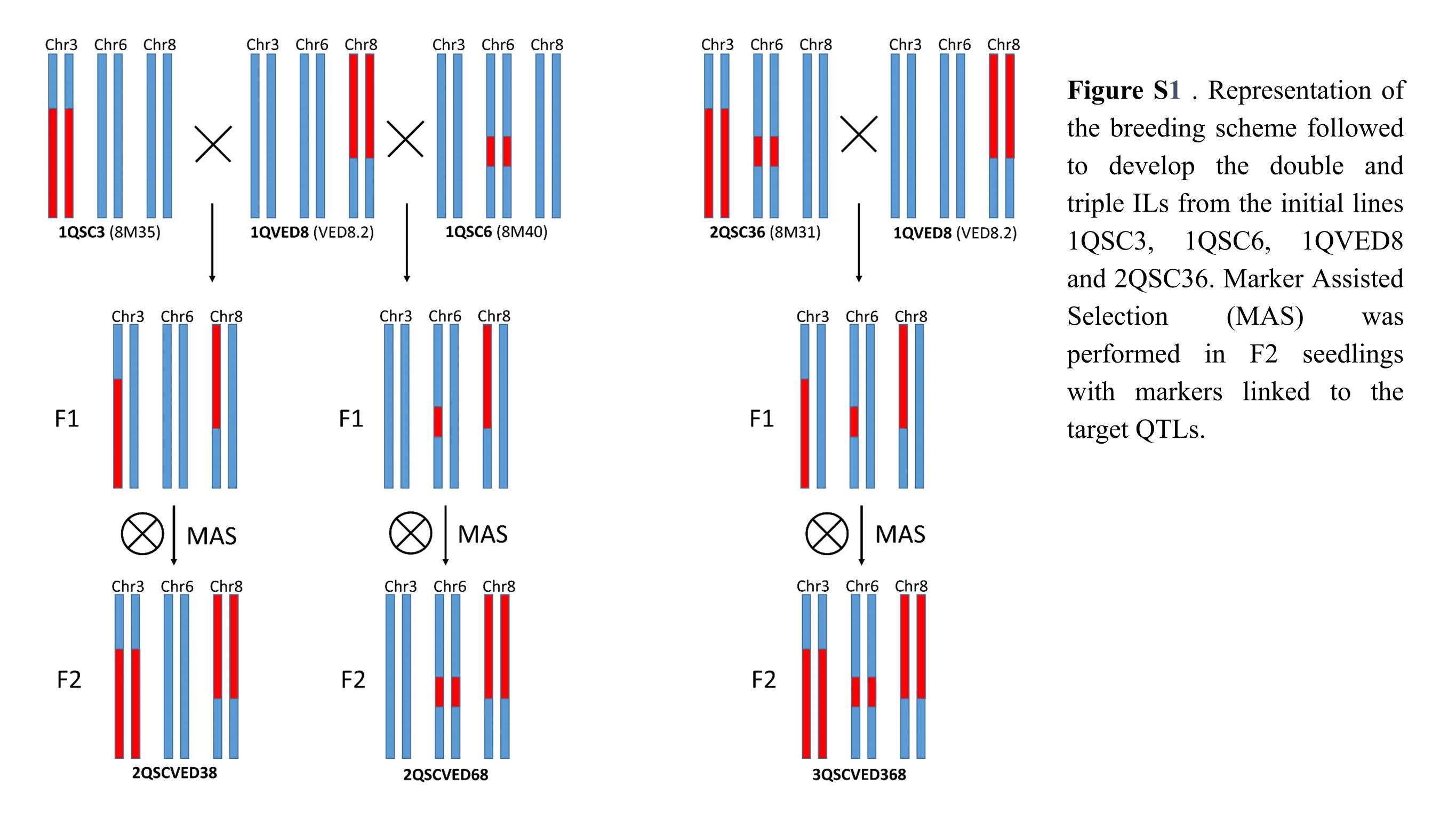

Supplement: Web_Material_uhac131 [file web_material_uhac131.zip › Figure S1.JPG]

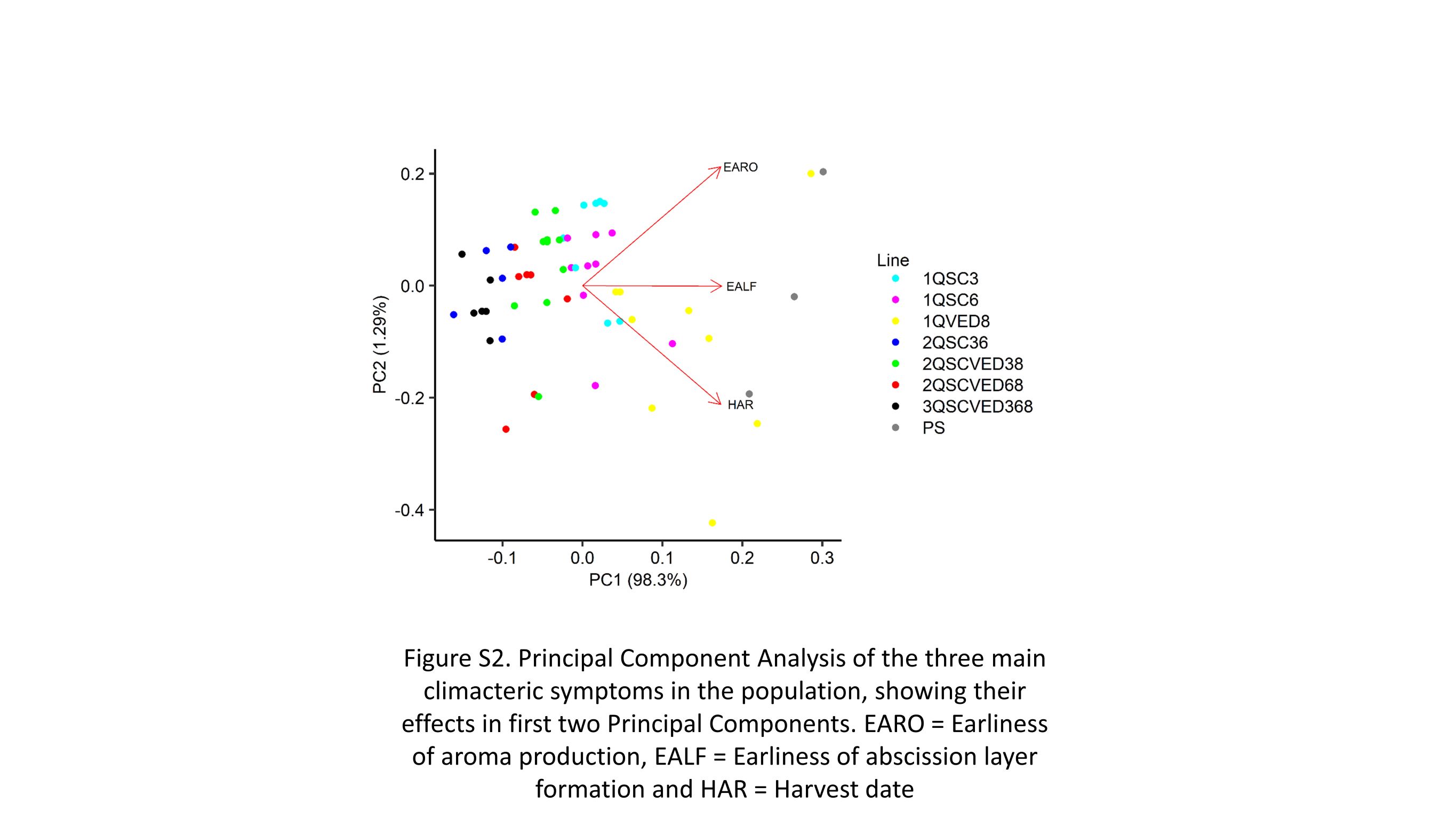

Supplement: Web_Material_uhac131 [file web_material_uhac131.zip › Figure S2.JPG]

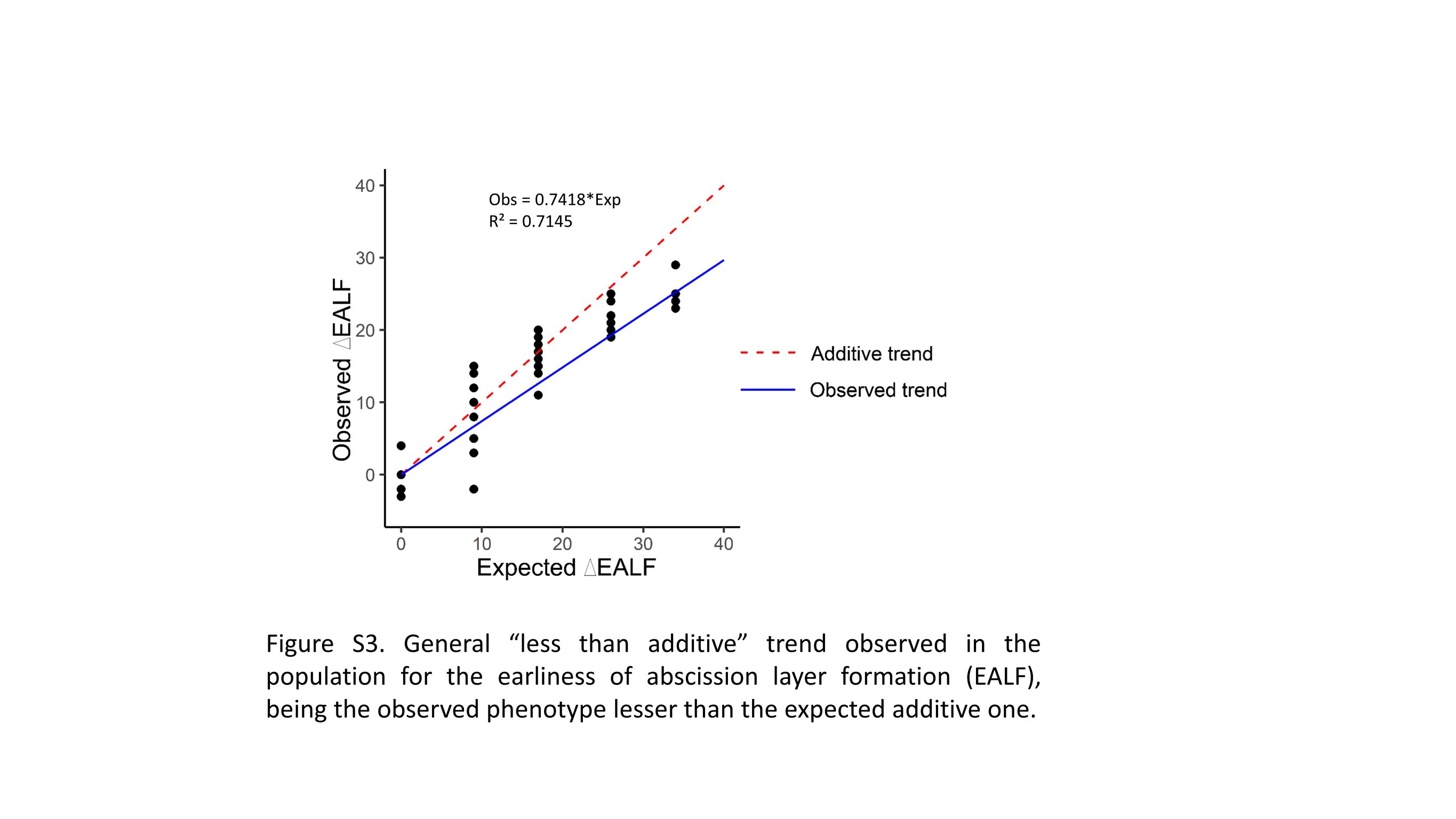

Supplement: Web_Material_uhac131 [file web_material_uhac131.zip › Figure S3.JPG]

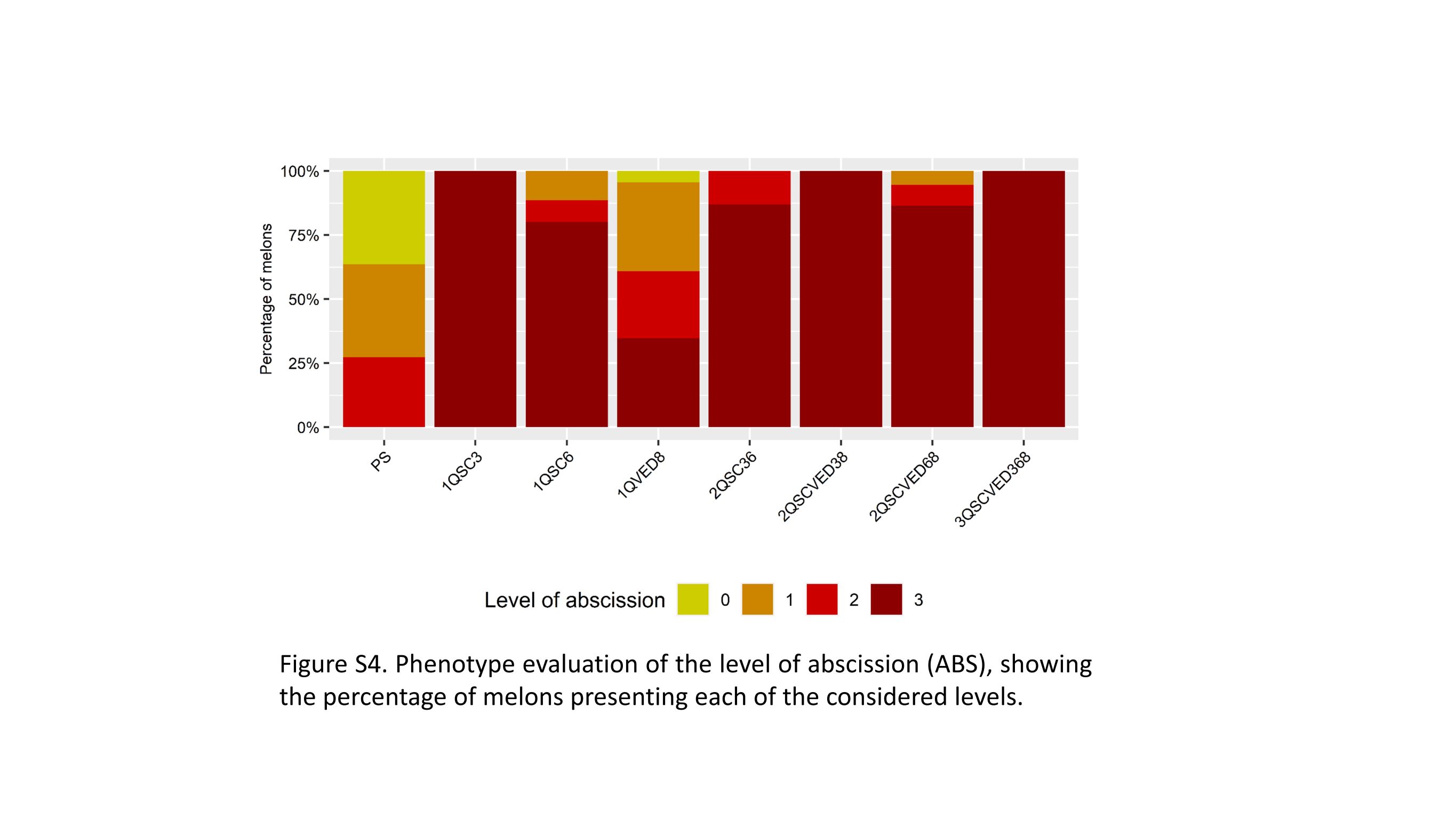

Supplement: Web_Material_uhac131 [file web_material_uhac131.zip › Figure S4.JPG]

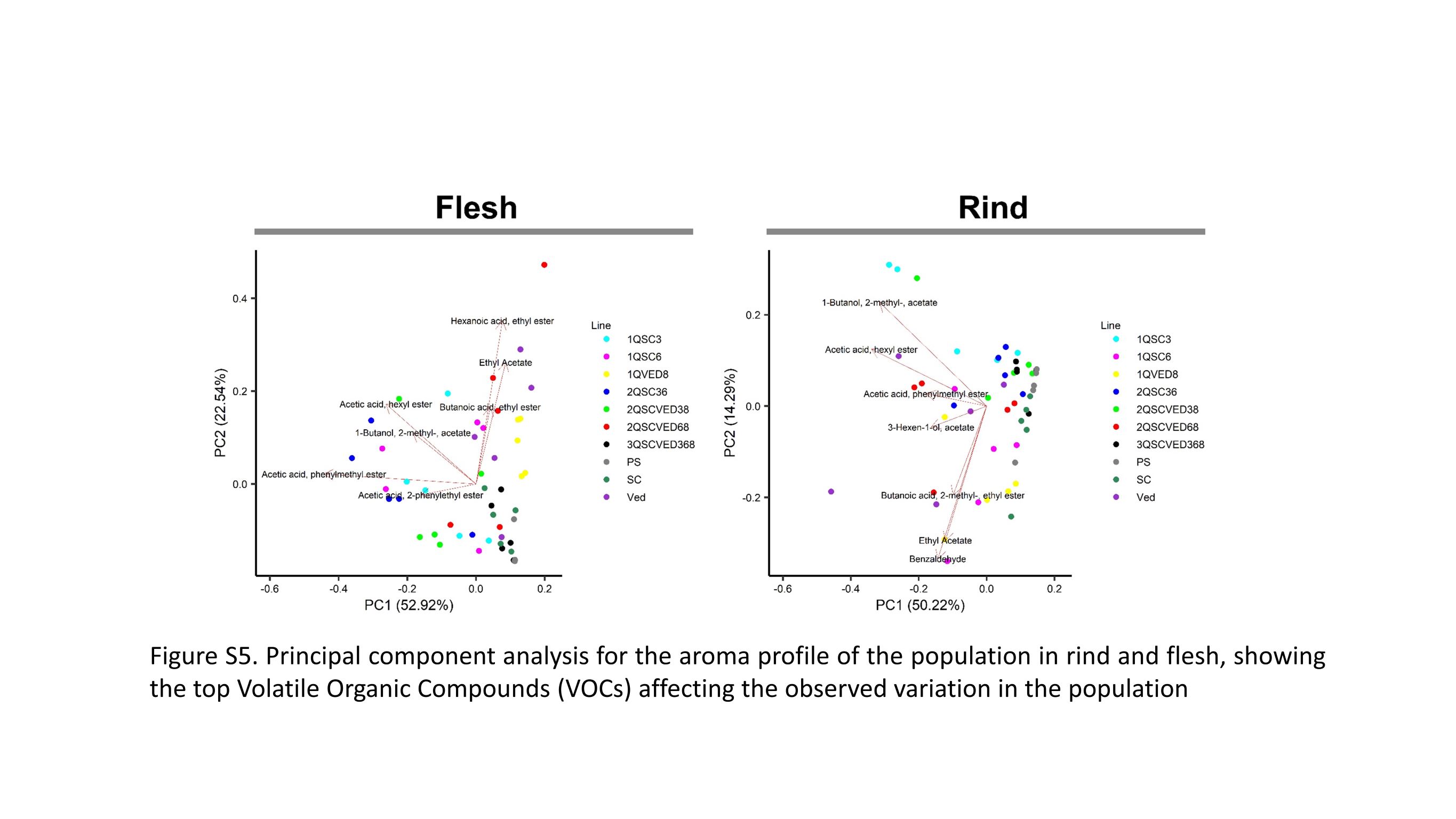

Supplement: Web_Material_uhac131 [file web_material_uhac131.zip › Figure S5.JPG]

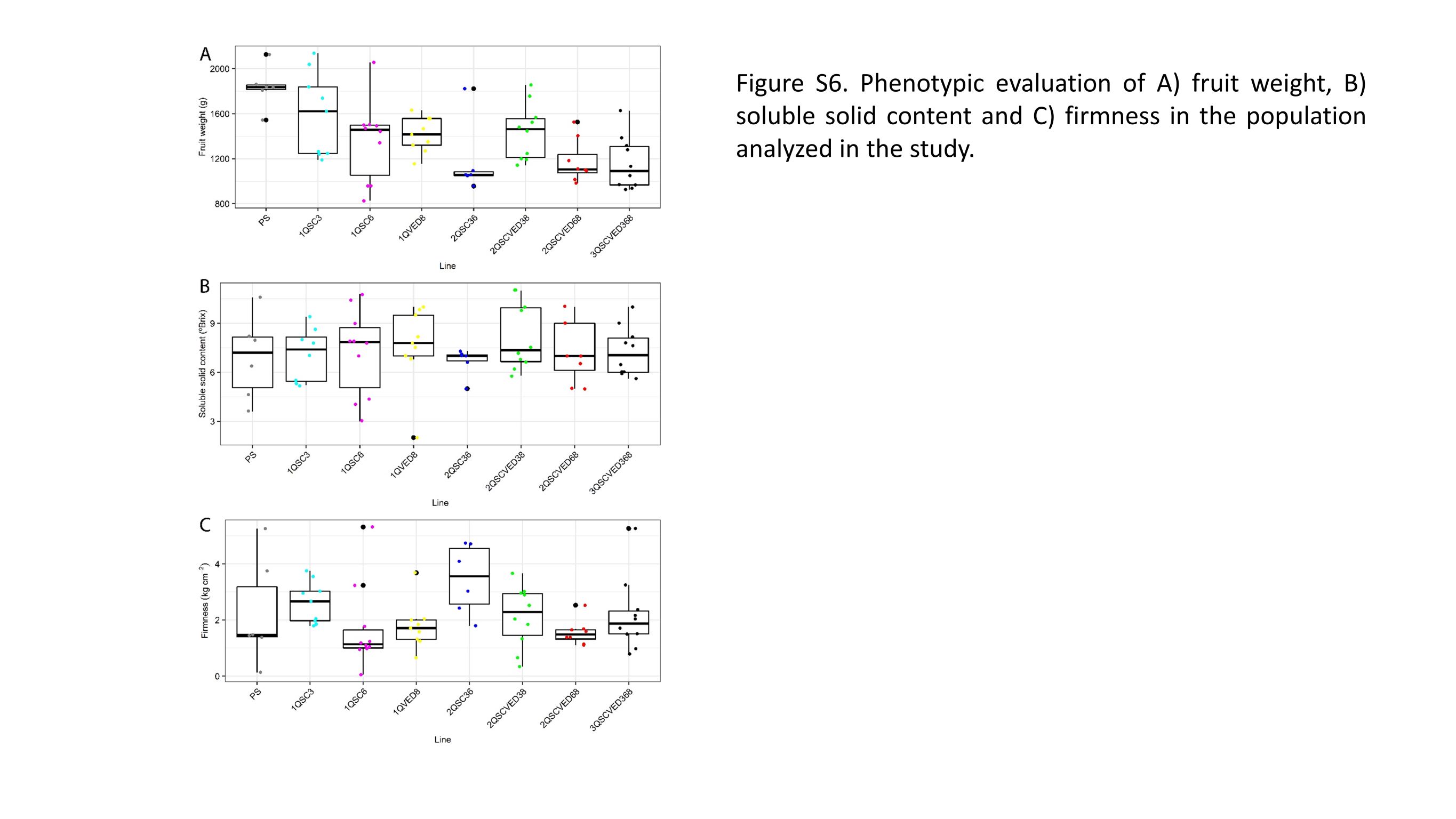

Supplement: Web_Material_uhac131 [file web_material_uhac131.zip › Figure S6.JPG]
